# The potential for artificial intelligence to predict clinical outcomes in patients who have acquired acute kidney injury during the perioperative period

**DOI:** 10.1186/s13741-021-00219-y

**Published:** 2021-12-15

**Authors:** Barry J. Kelly, Julio Chevarria, Barry O’Sullivan, George Shorten

**Affiliations:** 1 Department of Anaesthesiology and Intensive Care Medicine, University College Cork School of Medicine, Cork, Ireland; 2 Department of Nephrology, University College Cork School of Medicine, Cork, Ireland; 3 Insight Centre for Data Analytics, School of Computer Science & Information Technology, University College Cork, Cork, Ireland; 4 Anaesthesiology and Intensive Care Medicine, School of Medicine, University College Cork, Cork, Ireland

## Abstract

Acute kidney injury (AKI) is a common medical problem in hospitalised patients worldwide that may result in negative physiological, social and economic consequences. Amongst patients admitted to ICU with AKI, over 40% have had either elective or emergency surgery prior to admission. Predicting outcomes after AKI is difficult and the decision on whom to initiate RRT with a goal of renal recovery or predict a long-term survival benefit still poses a challenge for acute care physicians. With the increasing use of electronic healthcare records, artificial intelligence may allow postoperative AKI prognostication and aid clinical management. Patients will benefit if the data can be readily accessed andregulatory, ethical and human factors challenges can be overcome.

## Magnitude of the clinical problem

Acute kidney injury (AKI) is a common medical problem in hospitalised patients worldwide that can result in negative physiological, social and economic consequences. Acute kidney injury is estimated to complicate 12% of hospital admissions in the USA, directly affecting more than 2 million patients per annum (Al-Jaghbeer et al., 2018). According to the American College of Surgeons National Surgical Quality Improvement Program, complications caused by AKI occur in approximately 1% of all general surgery cases, resulting in an eightfold increase in all-cause 30-day mortality (Kheterpal et al., 2009). The AKI-Epi study showed that 57.3% of all ICU patients had AKI during a 1-week period. Amongst ICU patients with AKI, 44% had elective (29%) or emergency surgery (15%) prior to admission (Hoste et al., 2015). AKI following major abdominal surgery occurs in 13.4% of patients and is associated with a 12.6-fold (95% CI, 6.8–23.4) increased relative risk of short-term perioperative mortality (O’Connor et al., 2016). AKI occurs after 22% (median) of cardiac surgical procedures with 3% requiring renal replacement therapy (RRT) (Vandenberghe et al., 2016).

Patients who sustain AKI that requires dialysis (AKI-D) represent the severe end of the SA-AKI spectrum. AKI-D is an acute medical emergency that affects up to 13% of critically ill patients (Hoste et al., 2015). Approximately 50% of patients with AKI-D will not survive to hospital discharge, and a further 7% of survivors will remain dialysis-dependent at the time of hospital discharge and beyond (Wang et al., 2019). Even amongst those who survive and in whom dialysis can be discontinued, many have new or more severe chronic kidney disease (CKD) and 2-year survival from the time of hospital discharge amongst those with AKI-D is estimated to be 45–59% (Wonnacott et al., 2014).

The prediction and prevention of AKI during the intraoperative period has been reviewed extensively (Gumbert et al., 2020; Meersch et al., 2017). However, the requirement of a patient with AKI for dialysis represents an important categorical determinant of their overall outcome (Wang et al., 2019; Wonnacott et al., 2014). Estimating the likelihood of the need for RRT and subsequent outcomes at the time that AKI is diagnosed represents an important challenge for intensivists. This editorial focuses on the application of machine learning (ML) and artificial intelligence (AI) to big data to predict outcomes in patients who have already acquired new AKI during the perioperative period. We will discuss the limitations of current approaches to prognostication after AKI development and the potential role of, and barriers to, the use of ML/AI for the purpose of outcome prediction.

## Current approaches to AKI prognostication and their limitations

Predicting outcomes after AKI is difficult and the decision on whom to initiate RRT with a goal of renal recovery or predict a long-term survival benefit still poses a challenge for acute care physicians. Observational studies indicate that baseline estimated glomerular filtration rate (eGFR), proteinuria, greater age and diabetes mellitus are key factors associated with a lesser likelihood of recovery (Wonnacott et al., 2014). A systematic review of studies attempting to predict mortality after AKI highlighted 10 common variables that were frequently included in the prediction models, namely mechanical ventilation, age, gender, hypotension, liver failure, oliguria, sepsis/septic shock, low serum albumin, low level of consciousness and low platelet count (Ohnuma & Uchino, 2017). In a comprehensive meta-analysis looking at adverse events after AKI that included 82 eligible studies and 2,017,437 patients, of whom 255,264 (12.7%) developed AKI, male sex, baseline eGFR, coronary heart disease and diabetes were effect modifiers of the association between AKI and mortality. As would be expected, the risk of end-stage kidney disease (ESKD) increased with increased severity of AKI, as did ICU mortality. Patients undergoing angiography procedures and those with stage 3 AKI were at greatest risk of mortality (See et al., 2019). A recent meta-analysis identified that duration of AKI was independently associated with long term mortality, cardiovascular events and development of CKD stage 3 (Mehta et al., 2018).

Predicting outcomes in patients who have developed new AKI in the perioperative period poses a significant challenge. This is reflected in the limited number of studies in this area (Srisawat et al., 2011; Lee et al., 2019; Hoste et al., 2020). Primary outcomes prediction may include the prediction of those who go onto require dialysis, remain dialysis dependent or become liberated from the requirement for dialysis. Secondary outcomes may include 90-day mortality or 1- and 2-year mortality. A small study (*n* = 76), primarily assessing the role of urinary biomarkers to prognosticate for recovery, found that a lesser Charlson comorbidity index and Acute Physiological and Chronic Health Evaluation II (APACHE-II) score were predictors of recovery. The biomarkers of kidney injury explored in this study were urinary neutrophil gelatinase-associated lipocalin (uNGAL), urinary hepatocyte growth factor (uHGF), urinary cystatin C (uCystatin C), IL-18 and neutrophil gelatinase-associated lipocalin/matrix metalloproteinase-9 (Srisawat et al., 2011). Another study (*n* = 2 214) of AKI-D patients in the Kaiser Permanente Northern California electronic health record (EHR) system, derived a prediction model using age, chronic liver disease, preadmission CKD stage and haemoglobin concentration to predict likelihood of recovery of renal function (Lee et al., 2019). Recently, a prospective, multinational, observational study compared the predictive performance for persistent stage 3 AKI (KDIGO) of C-C motif chemokine ligand 14 (CCL14), a novel biomarker for AKI, with established biomarkers. Included in the comparison group were cystatin C, proenkephalin, NGAL and L-FABP. The AUC (0.83) for urinary CCL14 was significantly greater than for all other biomarkers. This novel biomarker of AKI may be useful to incorporate into future models to predict recovery from AKI (Hoste et al., 2020; Zarbock et al., 2018). In the RenalRIP trial, preoperative dickkopf-3 (DKK3), a renal tubular stress marker, was examined for utility of preoperative identification of patients at risk for AKI and subsequent kidney function loss in 733 cardiac surgery patients. DKK3 to creatinine concentrations higher than 471 pg/mg were associated with a significantly higher risk for AKI (OR 1.94, 95% CI 1.08–3.47, *p* = 0.026), persistent renal dysfunction (OR 6.67, 1.67–26.61, *p* = 0·0072), and dialysis dependency (OR 13.57, 1.50–122.77, *p* = 0.020) after 90 days compared with DKK3 to creatinine concentrations of 471 pg/mg or less (Schunk et al., 2019).

SA-AKI may result from hypovolemia, venodilation due to anaesthetic agents and positive pressure ventilation which may impair venous return. Other factors associated with increased rates of perioperative AKI include intraperitoneal open surgery, intraoperative blood transfusions, haemodynamic instability, intraoperative diuretics and use of vasopressors (Meersch et al., 2017).

## Artificial intelligence and machine learning for AKI prognostication

Artificial intelligence (AI) has a long history in the world of healthcare. MYCIN was one of the very early expert systems for treating blood infections (Shortliffe & Buchanan, 1975). Developed in the 1970s, the system had the ability to not only make diagnoses, but could also explain its reasoning. The system had a level of competence similar to that of human specialists. Expert systems are still in use today but tend to be described as rule-based systems or business rules systems, and they are normally hand-crafted by writing explicit rules that capture domain-specific expert knowledge (Zhao et al., 2010). A major advantage of rule-based AI systems is that they can readily explain their reasoning to human users and are easy to correct, update and maintain.

One of the most significant developments in AI over the past 20 years has been the rapid advancements in machine learning and, in particular, deep learning (LeCun et al., 2015). These have become amongst the most commonly used AI techniques in healthcare, demonstrating extraordinary levels of accuracy. For example, expert-level classification of skin cancer has been reported (Esteva et al., 2017).

However, the inherent complexity of AI may represent a barrier for clinicians to develop a trustworthy relationship with novel AI based tools. Classic linear methods of inference such as multivariable regression remain very attractive to clinicians. Knowledge of input, output and ‘everything in between’ facilitates transparency, reproducibility and reassures proponents of expert opinion of how the projections came to be. Despite the accuracy of deep-learning based approaches, the lack of ability to explain their reasoning to users has proved a challenge to a wider acceptance of AI in healthcare. This has led to the search for Explainable AI, or XAI. Explainable AI in which some visibility is provided to users of the mechanism underlying a prediction may be particularly valuable in supporting clinicians to use the outputs of AI/ML tools (Fellous et al., 2019). An interesting AI technique that has been recently applied to chronic renal disease is case-based reasoning (CBR) (Elkader et al., 2018; Tahmasebian et al., 2016; Vásquez-Morales et al., 2019). CBR has many distinct advantages in this setting. First, the technique does not attempt to generalise from case history. Instead, a classification of the current case, or a prediction of future outcomes, is made based on similar cases that have been experienced in the past. This is a natural approach to applying experience: the clinical view on the current situation is based on historical experience. Therefore, one achieves a number of interesting properties: diagnoses are easily explained since the similar cases that represent the wisdom applied to the current case directly provide a basis for explanation (Doyle et al., 2004). The number of similar cases and other properties of the neighbourhood of the current case provide a measure of confidence. The case-base of historical cases itself provides a notion of competence of the system, and one can also easily evaluate whether new experiences add value to the system (Smiti & Elouedi, 2014). While CBR systems might have the same accuracy levels and can often be comparable to deep learning systems in particular settings, their transparency makes them particularly interesting in clinical decision-making, especially since they essentially automate the natural reasoning of medical experts.

There are number of hurdles that must be overcome in order to actualize the full potential of AI as a prognostic tool. An inherent barrier to conducting longitudinal studies to predict outcomes following established postoperative AKI is the loss of follow-up in the postoperative period. Specifically, there is loss of follow-up at a human level, namely the perioperative physician, but also loss of data capture in the subsequent postoperative hours and days when subclinical renal injury occurs. The intraoperative period should represent the ideal scenario to capture an abundance of granular data that in turn could be used, firstly to predict AKI and of greater relevance here to construct outcome prediction models for outcome after AKI is diagnosed. Despite the more widespread use of anaesthesia information management systems (AIMS) to input patient demographics, chronic medical conditions, anaesthesia and surgical procedures, pharmacological interventions and physiological trends, there remains a paucity of studies using these data to enhance the care delivered to patients who develop perioperative AKI. One reason for this scarcity of research is that the current diagnostic standard for AKI, namely the Kidney Disease Improving Global Outcomes (KDIGO) guidelines has limitations in diagnosing AKI in the perioperative period. Urine output often decreases during the intraoperative period, due to release of aldosterone and vasopressin associated with stress, hypovolemia or even anaesthesia (Hahn & Warner, 2010). Studies using KDIGO frequently omit urine output as a criterion due to inability to accurately capture the data (Churpek et al., 2020). Using an increase in serum creatinine, as diagnostic criterion is also problematic because > 50% loss of renal function is required, before a resulting increase serum creatinine is observed. Additionally, there is an estimated time lapse of approximately 48 h between renal insult and an associated increase in serum creatinine. This phenomenon constitutes a renal ‘blind spot’ (Uchino, 2010). In time, we may see the role of novel biomarkers of renal injury and recovery, such as CCL14 being incorporated in future deep-learning approaches. However, unless the patient is in a care setting that allows continuous electronic monitoring, large quantities of potentially useful data points will continue to be lost.

Another barrier to the development of accurate prediction models is the difficulty in merging data from the perioperative period to the ICU/surgical ward and subsequently to nephrology outpatients and chronic dialysis units. The merging of data from a detailed preoperative assessment with high-fidelity data from the intraoperative period to include the post-operative care unit would at the very least enable the calculation of AKI incidence and frequency of initiation of renal replacement therapy. Data repositories that collected meaningful endpoints, to include those who progress to end-stage kidney disease (ESKD) at 90 days and mortality data would allow meaningful longitudinal data analysis. However, even in the era of widespread EHRs, renal outcome research relies on chart review by experts in the field (Wonnacott et al., 2014).

There are other challenges to introducing AI solutions into a clinical setting. While the focus in the literature tends to focus on achieving maximum diagnostic accuracy, there are many other issues to be considered. These include but are not limited to sensitivity (proportion of positives correctly classified), specificity (proportion of negatives correctly classified), false negative rate, false positive rate, the transparency and explicability of a decision. The use of AI at any point along the AKI casual pathway may allow earlier interventions that could improve the patient’s outcome. As the model continually learns from new data, the ideal goal would be time-updated risk stratification and prognostication. This has been attempted using electronic alerts to trigger a consultation from a nephrologist with mixed results (Colpaert et al., 2012; Wilson et al., 2021). However, given the low incidence rate (< 10%) of AKI and the potential of a low positive predictive value, such alerts may result in stretching the capacity of nephrology services beyond what is reasonable, offsetting any potential benefit to patients (Goldstein & Bedoya, 2020). Looking beyond the perioperative period, it is possible that AI can help guide appropriate outpatient follow-up and monitor for a long-term renal recovery after AKI. Recent literature reports of patients who recover from an AKI event, suggest that these patients have an increased risk of developing chronic kidney disease (Farooqi & Dickhout, 2016; Forni et al., 2017). This raises the prospect that an AI-led system could be used to follow-up AKI survivors.

There are challenges to testing or validating new prediction models across different healthcare systems and jurisdictions. Due to restrictions on the international transfer of data and General Data Protection Regulation (REGULATION (EU), n.d.). for example, studies are often limited to a single legislative jurisdiction. Research involving multi-jurisdictional Electronic Healthcare Records (EHRs) is hampered by challenges in coordination, ethical consent, differing protocols and costs. Traditionally, there has been some reluctance to creating and maintaining open source databases. This is particularly true in health systems in which EHRs were constructed primarily for coding of diseases and billing of services. There is an understandable reluctance to make these data widely available. In the past decade, there has been a move towards de-identified Open Data repositories (Martin et al., 2014). These can enable data generated by clinical studies to be reanalysed, re-interpreted or aggregated. The degree to which granular data can be easily assessed is crucial to the development of AI research in clinical medicine. Broadly, Open Data offers certain advantages: firstly, they provide transparency which enables audit and accountability; this includes identification of outliers of poor performance which may require targeted intervention. Secondly, they provide an invaluable resource that can drive innovation. The availability of Open Data can empower citizens and support clinicians, care providers and researchers to make better decisions, prompt new developments and identify inefficiencies, while ensuring that personal data remains confidential. The critical care community has begun to develop Open Access (largely structured) datasets which offer rich potential for AI applied to clinical questions (Johnson et al., 2016; Li et al., 2019; Faltys et al., 2020; AmsterdamUMCbd, n.d.) “(https://ec.europa.eu/info/sites/info/files/commission-white-paper-artificial-intelligence-feb2020_en.pdf)”. It is critically important that AI systems used in a healthcare setting are trustworthy. The European Commission has worked extensively on the notion of human-centric AI, and its High-Level Expert Group on AI developed a set of Ethics Guidelines for Trustworthy AI “(https://ec.europa.eu/info/sites/info/files/commission-white-paper-artificial-intelligence-feb2020_en.pdf)”.

From where would these big data repositories receive their data, and what would machine learning algorithms look like? The performance of any algorithm is only as good as the data that are imputed. For successful application of machine learning techniques to predict clinical outcomes, an ability to capture and merge all data acquired at pertinent time points along the renal injury pathway as described above would be required. Specific to perioperative AKI, the data management system would need to capture and merge clean data from preoperative assessment clinics, intraoperative anaesthesia information management systems (AIMS), postoperative care units, intensive care units and the hospital EHRs up to and including renal outpatients and chronic dialysis units. Presently, only the most advanced health care networks would have the ability to capture such granular data. An example of the use of AI to forecast an event, albeit AKI occurrence, is the large study by Tomasev et al. of 192 hospitals within the VA Health System, USA. Using data from 703,782 patients, the investigators divided the data into training (80% of observations), validation (5%), calibration (5%) and testing (10%) sets. The training set was used to train the proposed models. The validation set was used to iteratively improve the models by selecting the best model architectures and hyperparameters. The models selected on the validation set were recalibrated on the calibration set in order to further improve the quality of the risk predictions. The best models were finally evaluated on an independent test set that was withheld during model development. Using variations of recurrent neural network (RNN), the model predicts 55.8% of all inpatient episodes of acute kidney injury, and 90.2% of all acute kidney injury that requires subsequent administration of dialysis, with a lead time of up to 48 h and a ratio of 2 false alerts for every true alert (Tomašev et al., 2019). Using a similar concept, it may be possible to predict those are less likely to have a favourable outcome (alive at 90 days) after AKI development, redirect goals of care and withhold dialysis in the first instance. AI in healthcare, using a composite of electronic data and biomarkers of renal injury, may also allow electronic phenotyping of patients with AKI. This would analogous to a biological passport of renal injury. For example, data clustering may identify subgroups of AKI patients that are more likely to require long term dialysis or have limited survival benefit. Again, this knowledge could aid more informed goals of care.

## The role of AI for clinical decision support in treating patients with AKI

It is possible to imagine that an AI/ML tool could support a clinical decision at any ‘branch point’ in a standard management algorithm. In general, the further downstream the algorithm applied, the greater amount of patient-specific data available, but fewer effective measures exist to influence overall outcome. Thus, the selection of the question of prediction target is important. Once AKI has been identified (for instance based on KDIGO criteria) (KDIGO, n.d.) postoperatively, a prediction model could target several important ‘actionable’ targets: in-hospital mortality, requirement for dialysis, CKD.

Broadly speaking, a clinician would consider that a decision relating to an individual patient which results in a course of action that benefits the patient as a ‘good decision’. This presents four important challenges (or constraints) to any prediction tool being considered for decision support:

1. Is the entity being predicted clinically meaningful (would it matter to the patient?)
2. Is the currently available means for reaching the decision improved by the new tool?
3. Is the outcome being predicted amenable to change using existing therapies or interventions?
4. Has the relevance (values of predictive indices) of the predictor to this patient been quantified?

These constraints pose important limitations to the value of AI/ML led prediction of outcome for patients who suffer AKI in the postoperative period. The definitions of AKI and ‘outcome’ currently in use are non-uniform. Simple linear methods applied to datasets of modest size already offer good predictive indices for many patients in the group of interest (at least those towards the extremes of risk) (Ohnuma & Uchino, 2017). The interventions to be made or withheld based on risk of mortality (for instance) are also limited (Gaudry et al., 2016; New Engl J Med, 2020).

How should an AI-based tool which estimates propensity of a future clinical event be incorporated into a clinical decision? The current healthcare model places responsibility for recommending a course of action firmly with the clinician (or clinical team). The clinician assimilates information from various sources (e.g. laboratory values, medical images, physical examination, consultation with the patient and family members) to offer an ‘expert view’. This model assumes that the doctor is seeking to act in the patient’s best interest, (not necessarily to ensure optimal use of available resources), that the clinician is an expert whose estimation of what will actually happen as a result of different treatments is good, in particular, that it is superior to the estimation that the ‘non-expert’ patient would make with the same information available and that the patient trusts the clinician (Stewart, 1995). The introduction of the new AI predictive tool poses questions regarding this model: does the tool supersede the clinician opinion for part of the decision making activity; does the clinician incorporate the AI tool’s output into their thinking as another useful piece of information to be assimilated; does the AI tool render both clinician and patient ‘non-expert’ for the purposes of its output and thereby move the patient to a more central or active role in arriving at a decision? If dual process theory describes accurately how a clinician arrives at a decision, then he/she employs intuition (first pathway) and critical thinking (second pathway) together (Croskerry, 2017). The predictive value of any novel tool will inform the latter; it is possible that the intuitive weighting or emphasis (first pathway) that a clinician applies to the value will be subject to bias based on her understanding of, or belief in the novel prediction tool.

## Conclusion

Providing real time accurate prognostication tools to aid in clinical decision support represents a promising area in AKI research. At the point at which new AKI is diagnosed in the perioperative period, much data is available which could be applied to prediction of a clinical outcome of interest. An accurate prediction of outcome at that point could inform individual clinical decisions and improve understanding of the natural history of AKI. It is not difficult to envisage a useful interaction between models that predict new AKI and those that predict outcome after AKI. Patients will benefit if the considerable data access, regulatory, ethical and human factors challenges can be overcome.

## Data Availability

Not applicable

## Abbreviations

AI: Artificial intelligence
AKI: Acute kidney injury
AKI-D: Acute kidney injury requiring dialysis
APACHE II: Acute Physiological and Chronic Health Evaluation II
AUC: Area under the curve
CBR: Case-based reasoning
CCL14: C-C motif chemokine ligand 14
CKD: Chronic kidney disease
DKK3: Dickkopf-3
eGFR: Estimated glomerular filtration rate
EHR: Electronic health record
ESKD: End-stage kidney disease
GDPR: General Data Protection Regulation
ICU: Intensive care unit
KDIGO: Kidney Disease Improving Global Outcomes
ML: Machine learning
RRT: Renal replacement therapy
SA-AKI: Surgery-acquired acute kidney injury
uCystatin C: Urinary cystatin C
uHGF: Urinary hepatocyte growth factor
uNGAL: Urinary neutrophil gelatinase-associated lipocalin
